# Sequential determination of free and total cyanide by flow injection

**DOI:** 10.1155/S1463924699000048

**Published:** 1999

**Authors:** Maria Angélica Bonadiman Martin, Edgard Moreira Ganzarolli, Arilson Lehmkuhl, Ivan Gonçlves de Souza, Roldão Roosevelt Urzêdo de Queiróz

**Affiliations:** 1 Centro Federal de Educação Tecnológica do Paraná (CEFET), C. P. 391 Medianeira PR CEP 85884-000 Brazil; 2 Departamento de Química Universidade Federal de Santa Catarina Florianópolis SC CEP 88040-900 Brazil

## Abstract

This study presents a flow injection system for the sequential determination of free (CN^-^) and total (CN^-^+ HCN) cyanide using a potentiometric method which employs two different processes for the determination of these two chemical species. The first process is based on direct detection of CN^-^ using an ion-selective electrode for cyanide. In the second process, the sample is mixed with acid, and the released HCN is transferred across a PTFE membrane. The flow system employs three solenoid valves, a gas diffusion chamber, an ion-selective electrode, a potentiometer, and a computer with an AID conversion card. A Turbo Pascal^®^ computer program automatically performs all the steps involved in data acquisition and processing. The standard deviation for the results obtained with the proposed method was 0.5%.


Journal of Automated Methods & Management in Chemistry, Vol. 21, No. 1 (January–February 1999) pp. 23–26

### Sequential determination of free and total cyanide by flow injection

**Maria Angélica Bonadiman Marin, Edgard Moreira Ganzarolli,[1] Arilson Lehmkuhl,[1] Ivan Gonçalves de Souza[1] and Roldão Roosevelt Urzêdo de Queiróz[1]**

*Centro Federal de Educação Tecnológica do Paraná (CEFET), C. P. 391, CEP 85884-000, Medianeira, PR, Brazil; [1]Departamento de Química, Universidade Federal de Santa Catarina, CEP 88040-900, Florianópolis, SC, Brazil*

*This study presents a flow injection system for the sequential determination of free ($CN^-$) and total ($CN^- + HCN$) cyanide using a potentiometric method which employs two different processes for the determination of these two chemical species. The first process is based on direct detection of $CN^-$ using an ion-selective electrode for cyanide. In the second process, the sample is mixed with acid, and the released HCN is transferred across a PTFE membrane. The flow system employs three solenoid valves, a gas diffusion chamber, an ion-selective electrode, a potentiometer, and a computer with an A/D conversion card. A Turbo Pascal® computer program automatically performs all the steps involved in data acquisition and processing. The standard deviation for the results obtained with the proposed method was 0.5%.*

### Introduction

Numerous works based on flow injection analysis (FIA) have been published lately [1]. Many of them [2–4] describe the use of loop-based injectors to introduce the sample into the carrier stream. A common way of changing the volume of the sample being introduced into the flow system is to change the length of the sampling loop. In other cases, three-way solenoid valves are used for sample introduction, and the system is triggered either by a built-in electronic circuit [5] or microcomputer [6]. Usually, in methods that employ manual injection, signals coming from the detection system are sent directly to a printer, generating a real-time diagram. In automated methods, a microcomputer recieves, processes and records the signals.

Many projects have been developed to monitor cyanide in waste waters [7]. One of the most commonly employed techniques is direct potentiometry with an ion-selective electrode (ISE) [8]. The classic method for determination of total cyanide in effluents is distillation, to eliminate interference, followed by colorimetric detection. This is an onerous method, since it requires expensive reagents, as well as several hours of laboratory work. Besides, it is used only for determination of low levels of cyanide, and it has the additional disadvantage of not being easily automated. Finally, this method does not allow quantification of free cyanide in the original sample.

To quantify free cyanide ($CN^-$) and total cyanide ($HCN + CN^-$), it is necessary to employ a non-destructive technique for determination of free cyanide, and a different technique for determination of total cyanide [9]. In this paper, we present an automated flow system which is capable of sequentially determining both free and total cyanide in aqueous samples. For the determination of free cyanide, we employ a technique of direct flow injection with potentiometric detection through an ion-selective electrode (potentiometric flow-through detection). For determination of total cyanide, we employ an acid-flow injection technique, followed by permeation across a gas-diffusion membrane and posterior potentiometric detection (gas-diffusion flow injection potentiometry).

In the system being proposed, these two techniques are implemented concurrently in a single flow system which employs three three-way solenoid valves, a gas diffusion chamber, an ion electrode for cyanide, and a reference electrode. This system performs the sequential determination of free and total cyanide using two distinct analytic curves.

### Experimental procedure

### Equipment

The analysis module consists of an Ismatec peristaltic pump (model 7341-15, fixed speed), equipped with Tygon® tubing with various internal diameters; polyethylene tubing (i.d. = 0.8 mm); three three-way solenoid valves (Cole Parmer); an Orion potentiometer (model 720A); a reference double junction Ag/AgCl electrode (Cole Parmer 5990-64); a cyanide-indicator (Orion 94-06BN); cell for adaptation of the indicator electrode to the flux system [10]; gas diffusion chamber [11]; analogic/digital MQI12/8PCC conversion card (Microquímica Ltda.); 486 DX2-66 microcomputer; and software developed in Turbo Pascal 7.0®.

### Solutions

All solutions were prepared with distilled or deionized water. For the cyanide stock solution ($1.0 \times 10^{-10}$ mol dm$^{-3}$), we weighed 0.65 g KCN. This was diluted in 50 ml water; 1 ml NaOH mol dm$^{-3}$ was added, and the volume was completed to 100 ml. From this solution, we prepared analytic solutions of $1.0 \times 10^{-5}$, $3.0 \times 10^{-4}$, $1.0 \times 10^{-4}$, $3.0 \times 10^{-3}$, and $1.0 \times 10^{-2}$ mol dm$^{-3}$. The pH for these solutions was measured daily, before the beginning of the experiments. pH determination was carried out with a glass electrode and a potentiometer with a 0.01 pH unit resolution.

The carrier solution was obtained by diluting the $1.0 \times 10^{-3}$ $CN^-$ analytic solution 100 times and by adjusting the pH with NaOH to 11.5.

1463-9246/99 $12·00 © 1999 Taylor & Francis Ltd

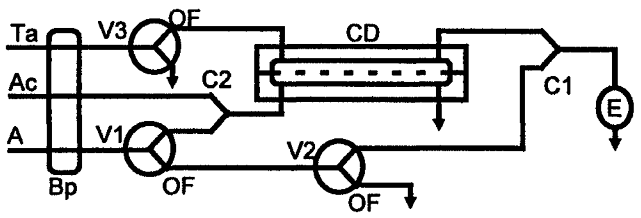

*Figure 1. Flow chart of the flow injection system. Sample line (A), transporter line (Ta) and acid line (Ac). 'E' is the potentiometric detector; C1 and C2 are the junctions, CD is the gas diffusion chamber with a PTFE membrane; V1, V2 and V3 are three-way solenoid valves.*

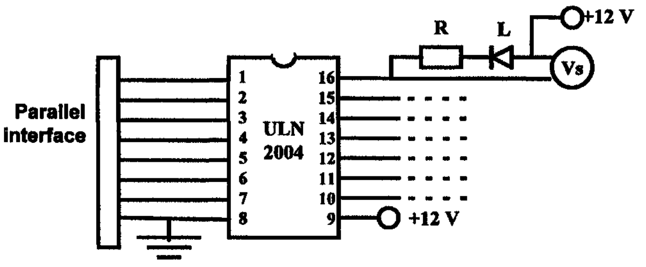

*Figure 3. Power drive and source for triggering the solenoid valves. The figure shows the 670 Ω resistor (R); a common LED (L); solenoid valves (Vs).*

### *Description of the process*

Figure 1 shows the flow chart for the determination of free and total cyanide. In the process of free cyanide determination, valves V2 and V3 are triggered simultaneously, so that the sample is directly injected into the carrier stream. Triggering of valve V3 causes interruption of the carrier stream. Triggering of valve V2 causes the sample to be introduced through junction C1. Therefore, when valves V3 and V2 are triggered, a sample zone is formed, whose volume depends on the triggering time interval of valves V2 and V3.

For determination of total cyanide, only valve V1 is triggered. The sample flows together with a sulphuric acid solution through junction C2. This causes liberation of HCN, which crosses a PTFE membrane in the gas diffusion chamber (CD). After having crossed the membrane, at the other side of the chamber, HCN is converted into $CN^-$ by the carrier solution (NaOH solution). The newly formed $CN^-$ is determined by the same electrode in both processes.

To quantify the two species of cyanide in a sample, it is necessary to produce two distinct analytic curves, one for the determination of free cyanide, and the other for determination of total cyanide.

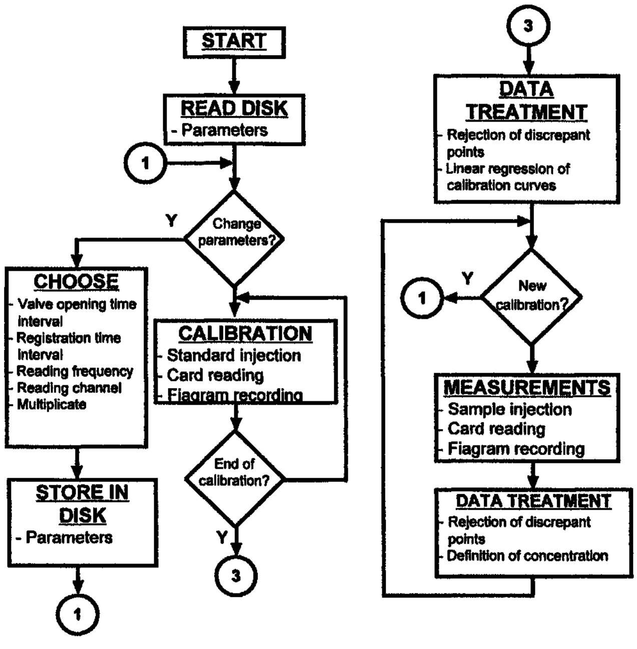

*Figure 4. Algorithm flow chart of the control software, data recording and treatment.*

### *Automation process*

The electric circuit is described in figure 2. A microcomputer sends control signals to the computer parallel interface. These signals are recieved by a home-made power drive (figure 3), which basically consists of a built-in power source and a ULN2004 integrated circuit. This drive triggers the solenoid valves (Vs). The signals coming from the electrode system are read by the potentiometer and sent to the analogic/digital conversion card, and then acquired by the software. At the same time, a data file is created and the diagram appears in the computer screen in real time.

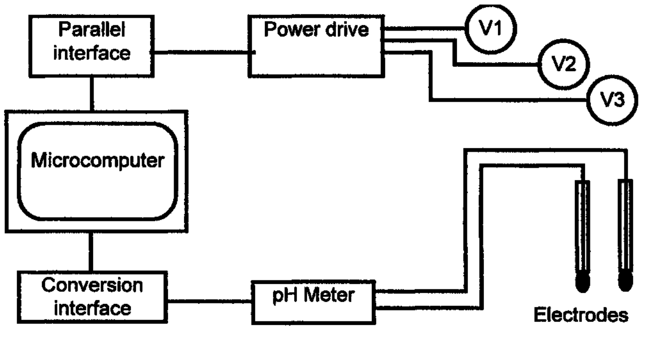

*Figure 2. Computed system for control of solenoid valves and reading of A/D conversion card.*

The program to control triggering of the valves, data acquisition and mathematical treatment of the data was developed in Turbo Pascal® 7.0 [6], as shown in the flow chart presented in figure 4. The basic reading and conversion routine performed by the AD card was developed in Assembler, and allows up to 20 000 readings per second. The time interval for valve opening can vary from 1 msec to 1 min. Throughout the experiment, after each reading, the converted value appears in the computer screen, and the final recording of all values appears as a diagram. Two data files (ASCII), one containing the diagram, and another containing the maximum values for each injection, are stored in the computer disk for later mathematical treatment.

*System performance assessment*

To assess system performance, we determined free cyanide, and HCN in synthetic samples, with different pH values and with the same total cyanide concentration, which was fixed as $10^{-4}$ mol dm$^{-3}$. The different pH values were used to produce a variation in the ratio between $CN^-$ and HCN concentrations. For that, we considered the HCN p$K_a$ in an aqueous solution (p$K_a$ = 9.14). The pH values necessary to obtain $[CN^-]/[HCN]$ rates equal to 1/100, 1/10 and 10/1 were calculated using equation (1):

$$pH = pK_a + \log [CN^-]/[HCN] \quad (1)$$

The calculations revealed that $pH_{(1/100)} = 7.14$; $pH_{(1/10)} = 8.14$ and $pH_{(10/1)} = 10.14$.

These same samples were submitted to determination using our proposed method and the traditional potentiometric method, so that results could be compared. HCN concentration was indirectly determined in both methods, since this value was obtained from the difference between total cyanide concentration and $CN^-$ concentration.

To compare the precision of the two methods, all determinations were replicated six times.

To perform the determination of the two species, HCN and $CN^-$, using the method proposed herein, we used three consecutive injections. The potential value taken into consideration was obtained from the average among the three resulting peaks. The injections performed in triplicate were also used to plot the two analytic curves.

### Results and discussion

Baseline stability and injection reproducibility are shown in figure 5, which represents the diagrams for potential versus time for the two processes carried out in the proposed method. After statistical evaluation, we obtained the following functions in relation to the analytic curve for processes 1 and 2, respectively: $Y_1 = 687.897 + 59.077\ X_1$; and $Y_2 = 615.465 + 51.595\ X_2$. These curves had a good linearity, confirmed by

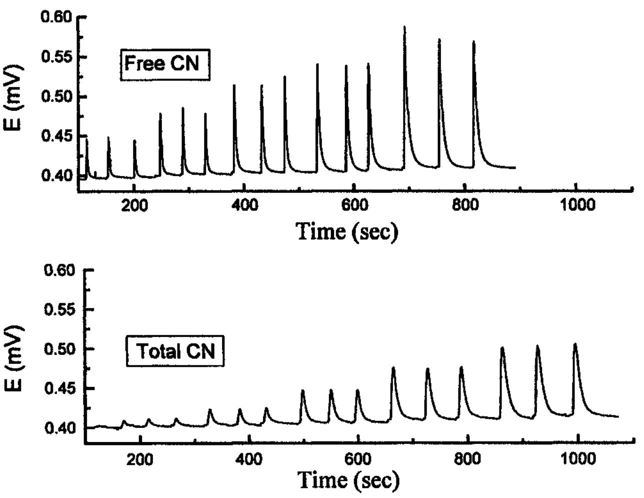

*Figure 5. Diagrams of the two processes, obtained with five standard solutions and injections performed in triplicate.*

their respective correlattion coefficients $r_1 = 0.998$ and $r_2 = 0.996$.

The lower detection limit was approximately $1.0 \times 10^{-5}$ mol dm$^{-3}$ for both processes. The injection system described in figure 1 presents a high level of stability and repeatability of the measurements, and can be applied for cyanide determination of industrial waste waters. Tables 1–3 show the results obtained in the experiments performed for assessment of equipment performance.

The comparison between $CN^-$ and HCN concentrations as determined by the two methods reveals good agreement in terms of the results for the three rates under study (tables 1–3). The percentile difference between these two methods can be seen in the last column of tables 1–3. For the three rates there was positive deviation in relation to $CN^-$ concentration, and negative deviation in relation to HCN concentration.

However, this deviation is not very significant, since, with the exception of HCN determination using the 10/1 rate, the deviation values are smaller than the standard deviation of the results. This suggests that the proposed method has a high degree of precision in comparison to

*Table 1. $CN^-$ and HCN concentrations at an approximate rate of 1/100 (pH = 7), determined with the traditional potentiometric method and with the proposed method (replicated six times for each method).*

| *Chemical species* | *Stationary method (mol dm$^{-3}$)* | *% SD* | *Proposed method (mol dm$^{-3}$)* | *% SD* | *% Difference between methods* |
|---|---|---|---|---|---|
| $CN^-$ | $(1.5 \pm 0.01)10^{-6}$ | 0.7 | $(1.58 \pm 0.02)10^{-6}$ | 1.3 | +0.764 |
| HCN | $(98.4 \pm 0.5)10^{-6}$ | 0.51 | $(98.4 \pm 0.5)10^{-6}$ | 0.5 | −0.012 |

*Table 2. $CN^-$ and HCN concentrations at an approximate rate of 1/10 (pH = 8), determined with the traditional potentiometric method and with the proposed method (replicated six times for each method).*

| *Chemical species* | *Stationary method (mol dm$^{-3}$)* | *% SD* | *Proposed method (mol dm$^{-3}$)* | *% SD* | *% Difference between methods* |
|---|---|---|---|---|---|
| $CN^-$ | $(1.13 \pm 0.01)10^{-5}$ | 0.9 | $(1.15 \pm 0.05)10^{-5}$ | 2.3 | +1.769 |
| HCN | $(8.87 \pm 0.04)10^{-5}$ | 0.45 | $(8.85 \pm 0.09)10^{-5}$ | 1.0 | −0.225 |

*Table 3. $CN^-$ and HCN concentrations at an approximate rate of 10/1 (pH = 10), determined with the traditional potentiometric method and with the proposed method (replicated six times for each method).*

| *Chemical species* | *Stationary method* ($mol\,dm^{-3}$) | *% SD* | *Proposed method* ($mol\,dm^{-3}$) | *% SD* | *% Difference between methods* |
|---|---|---|---|---|---|
| $CN^-$ | $(9.10 \pm 0.04)10^{-5}$ | 0.4 | $(9.12 \pm 0.08)10^{-5}$ | 0.8 | +0.219 |
| HCN | $(0.90 \pm 0.01)10^{-5}$ | 1.1 | $(0.88 \pm 0.01)10^{-5}$ | 1.1 | −2.444 |

the stationary method. Data analysis using the *t*-test resulted in a 95% confidence level.

### Conclusions

The main characteristic of the analytical procedure being proposed here is operational simplicity, since the sensor used for detection consists merely of a cyanide-indicator electrode.

The system responds efficiently and quickly, has a good level of repeatability (r.s.d. < 0.5%, $n = 6$), allows performance of 60 measurements per hour, and has good baseline stability (±1 mV). Also, our method is not significantly influenced by temperature; there is no need to temperature control if it does not vary significantly during measurements. The response linear range is wide (from $1.0 \times 10^{-5}$ to $1.0 \times 10^{-2}\,mol\,dm^{-3}$), with a lower detection limit of $8.0 \times 10^{-6}\,mol\,dm^{-3}$. The computer program necessary for data aquisition and data treatment can be obtained from the authors. These include source code write in Turbo Pascal 7.0 and executable codes for use with IBM-compatible computers.

### Acknowledgment

The authors wish to thank the CAPES and FINEP for financial support.

### References

1. Reis, B. F., 1996, *Quím. Nova*, **19**, 51.

2. Reis, B. F., Giné, , M. F., Kronka, E. A. M. and Eloisa, A. M., 1992, *Quím. Nova*, **15**, 231.

3. Reis, B. F., Martelli, P. B., Menegario, A. A. and Giné, M. F., 1993, *Quím. Nova*, **16**, 109.

4. Reis, B. F. and Bergamin, F. H., 1993, *Quím. Nova*, **16**, 570.

5. Faria, L. C. and Pasquini, C., 1991, *Quím. Nova*, **14**, 216.

6. Pasquini, C. and Faria, L. C., 1991, *Journal of Automatic Chemistry*, **13**, 143.

7. Cusbert, P. J., 1976, *Anal. Chim. Acta.*, **87**, 4299.

8. Figuerola, E., Florido, A., Aguilar, M. and Pablo, J., 1980, *Fresenius' Z. Anal. Chem.*, **331**, 620.

9. Riseman, J. H., 1972, *Am. Lab.*, **4**, 63.

10. Marin, M. A. B., Ganzarolli, E. M., Queiróz, R. U. and Souza, I. G., *Quím. Nova* (in press).

11. Durst, R., 1977, *Annal. Lett.*, **10**, 961.

